# Irrigation of the Urethra

**Published:** 1901-12-07

**Authors:** 


### IRRIGATION OF THE URETHRA.

Dr. Louis Broter[1] evidently exaggerates greatly when he says that the irrigation method of treating acute gonorrhœa "is one of the greatest blessings that human thought and observation have furnished to mankind." Still there may be some good in the treatment, notwithstanding the style in which it is set forth by its advocate. Dr. Broter first insists upon the necessity of obtaining complete asespis of all the nozzles and other instruments employed, and he recommends the use of certain appliances which are doubtless of considerable advantage in carrying out any such treatment, for example the wearing of an indiarubber apron by the patient, as he stands before the surgeon during the applications, the apron having a hole for the penis in the middle of it. The solution employed is one of permanganate of potassium. 1 in 4,000 increased up to 1 in 1,000 as the disease advances. Of this solution a quart is to be used at each application. Its temperature should be at least 100° F. and may be increased up to 112-120° F. as the patient becomes accustomed to it. The stream is directed into the meatus with gradual increase of force. "Should it be desired to wash out the posterior urethra or the bladder the nozzle must be brought into close proximity to the meatus, at the same time telling the patient to attempt to urinate. This last act, as will be readily understood, loosens the cut-off muscles and the solution gains an entrance. This is continued until an entire quart of the solution is used." After the irrigation three drachms of a 2 per cent. solution of protargol are injected through an ordinary urethral syringe. This solution is retained, if possible, for five or ten minutes, when it is allowed to flow away. The number of irrigations required varies. For the first two or three weeks they may have to be given twice a day, and then, when the discharge has changed from purulent to serous, they may be given once a day and followed by injections of zinc or lead, with or without bismuth, instead of the protargol injections. Even after the urine is clear and the discharge and gonococci have disappeared, the irrigations are to be continued once a week for another month. It is evident then that unless gonorrhœa is a very much more serious affair in New York than it is with us, this treatment does not offer any great advantage in regard to the time or the care required. Nor does it appear that even Dr. Broter trusts exclusively to irrigation, for not only does he employ the above-mentioned injections but he gives alkalies and diuretics, such as bicarbonate of soda and acetate of potash, while he finds methylene blue and boracic acid in pill three times a day a good diuretic and urinary antiseptic during the first two weeks, after which he gives "oleum santali, balsam copaibæ, or the various preparations of cubeb, in capsule form." Probably the irrigations are chiefly of service in washing away the pus and mucus from the lining membrane of the urethra; and if this can be satisfactorily done by a mere ascending urethral douche, without passing an instrument over the inflamed surface, it is easy to see the advantages of the method. It would appear, however, that the irrigations are hardly to be regarded as in themselves constituting the treatment, and that to speak of it as the "irrigation treatment" is not a very accurate use of words.

[1] New York Med. News, Nov. 16.

